# Interstitial magnetic resonance lymphography is an effective diagnostic tool for the detection of lymph node metastases in patients with cervical cancer

**DOI:** 10.1186/1471-2407-12-360

**Published:** 2012-08-18

**Authors:** Ying Hong, Luojun Xiang, Yali Hu, Zhengyang Zhou, Haiping Yu, Bing Zhu

**Affiliations:** 1 Nanjing Drum Tower Hospital, Nanjing Medical University, Nanjing, 210008, China; 2 Nanjing Drum Tower Hospital, The Affiliated Hospital of Nanjing University Medical School, No.321 Zhongshan Road, Nanjing, 210008, China; 3 Nanjing University of Chinese Medicine, Nanjing, 210029, China

**Keywords:** Cervical cancer, Magnetic resonance (MR), Lymphography

## Abstract

**Background:**

The aim of the present study was to determine the feasibility of detecting sentinel lymph node (SLN) metastases using interstitial magnetic resonance (MR) lymphography in patients with cervical cancer. MR data were compared to pathological results from the lymph nodes excised during surgery.

**Methods:**

Twenty-eight patients with cervical cancer were enrolled and studied from January 2006 to December 2010. All patients underwent interstitial MR lymphography to determine the presence of sentinel lymph nodes and visualize lymphatic vessel drainage in the pelvis. Radical hysterectomy and excision of pelvic lymph nodes was performed according to their lesion grade. Gadodiamide was injected either intradermally into the bipedal toe web, into the labia majora or into the cervical tissue. MR results were compared with pathological reports.

**Results:**

In 28 patients, lymphatic vessel drainage and lymph node groups were clearly visualized. Of these, 5 were MR lymphography positive and 23 were MR lymphography negative. Six had pathologically proven metastasis, five had true positives and 1 had a false negative in the obturator lymph node.

**Conclusions:**

Interstitial MR lymphography can be used to determine the extent and shape of pelvic lymphatic vessel drainage and lymph node metastases in patients with cervical cancer.

## Backgound

The incidence of cervical cancer has increased significantly, particularly in younger women [1,2]. However, the treatment for cervical cancer, along with the preservation of the patients’ organs and physiological function as much as possible, remains a clinical challenge.

Traditional lymphography technology used during surgery cannot predict lymph node metastasis of cervical cancer before the operation. Interstitial lymphography avoids trauma to the lymphatic system experienced in direct lymphography and can display lymph channels and lymph nodes [3]. Using albumin-Gd-DTA as a contrast agent, they were able to classify regions of interest as ‘pooling’ if the macromolecular contrast media concentration increased over time, or ‘draining’ if it decreased relative to early phase images. The more invasive tumor line had a significantly higher number of draining voxels by MRI. Thus, lymphatic drainage patterns correlate with the rate of metastasis and lymphangiogenesis. Drainage may be dependent on both the invasiveness of the tumor and on the extracellular matrix integrity, which, if reduced, can facilitate the passage of tumor cells along with extracellular fluid.

The purpose of the present study was to evaluate the diagnostic role of interstitial MR lymphography for the detection of lymph node metastases, which in combination with the patients’ other clinical characteristics (e.g. tumor size) will help to better define a treatment strategy.

## Methods

Twenty-eight patients with cervical cancer who had undergone uterine surgery between January 2006 and December 2010 were included in this study. The patients were between 35 and 76 years old. The cervical tumor types included 26 squamous cell carcinomas and two adenosquamous carcinomas. The study was based on the statement of ethical principles in the Declaration of Helsinki. Accordingly, the study was reviewed and approved by the clinical research ethics committee of the Affiliated Drum Tower Hospital of Nanjing University Medical School (Approval Number 2010028) and signed consent was obtained from each patient.

All patients were scanned using interstitial MR lymphography to examine lymphatic vessel drainage and lymph nodes prior to pelvic surgery. Surgery included radical hysterectomy and removal of pelvic lymph nodes according to their lesion grade. Pathological diagnoses were based on the analysis of the excised lymphatic tissue.

Inclusion criteria for this study were as follows:

(1) cervical cancer, stage Ia2-IIa;

(2) no prior pelvic surgery;

(3) no birth control ring;

(4) signed consent for MR lymphography.

Patients with contraindications to MR imaging and patients with contrast agent allergies were excluded. The patients were admitted eight hours prior to the exam, and received a clysma two hours before MR lymphography. Initially, Gadodiamide was injected intradermally into the bipedal toe web (four cases) to evaluate image quality before the study was continued using different injection locations. Subsequently, Gadodiamide was injected into the labium majus in two cases and into the cervical tissue in 22 cases to evaluate the sentinel lymph node (SLN) of cervical tissue and lymph node metastases. Patients were injected with 12–20 ml of Gadodiamide (body weight adjusted dose) plus 4 ml Lidocaine (2%) within 5 minutes. Gadodiamide was injected with a 0.20-0.25 ml/kg body weight (60 kg - 80 kg) adjusted dose leading to injected contrast agent volumes of 12–20 ml. The tissue close to the injection site was then massaged for about 30 seconds. After contrast injection, Mupirocin ointment (main components: Mupirocin, ointment matrix polyethylene glycol) was applied to the injection location to prevent infection.

MR imaging was performed immediately following administration of Gadodiamide. The MR exams were performed in the supine position on a Philips Intera 1.5 T MR system and a Philips Achieva 3.0 T TX MR system with a quadrature body coil. The following MRI sequences were used for MR lymphography of the pelvis:

1. T2-weighted(T2W)turbo spin-echo (TSE) in the axial (ax, TRA) plane;

2. T1W_TSE_ax, T2W_SPAIR coronal (COR);

3. T2W_ SPAIR sagittal (SAG);

4. PDW_ SPAIR TRA, DWIBS TRA.

Repetition Time (TR) and Echo Time (TE) were 5.6 ms and 1.86 ms, respectively. Each of the sequences included a slice thickness of 0.5 mm and 300 slices. Gadodiamide was injected into each patient within 5 minutes. Subsequently, a 3D MR lymphography sequence in the renal plane was begun after 3, 6, 9, 12, 15, and 20 minutes. In cases where the contrast injection was incomplete, the scan was repeated until completion. The MR lymphography sequence had a slice thickness of 0.5 mm and 300 slices with a TR = 5.6 ms and TE = 1.86 ms. Subsequently, the following MR sequences were used post-contrast:

1. T1W_TSE_ax TRA,

2. T1W_TSE_ax SAG,

3. T1W_TSE_ax COR.

The following diagnostic classifications were used in the analyses of the MR images: Lymphatic metastases:

1. Positive when lymphatic vessels were discontinuous in shape or were lymph nodes with filling defect;

2. Negative when lymphatic vessels and lymph nodes were regular in shape.

Patients with cervical cancer of stages Ia2, Ib or IIa were analyzed for lesion location and the extent of lymph node involvement using interstitial MR imaging and had radical abdominal hysterectomy and pelvic lymph node dissection following interstitial MR imaging. The pathological results were compared to the results of lymphatic MR imaging.

## Results

Twenty-eight patients with cervical cancer who had undergone uterine surgery between January 2006 and December 2010 were included in this study. Of the 28 cervical cancer patients who were scanned with MR lymphography, there were 5 MR lymphography positive and 23 MR lymphography negative. One case with an MR positive result in the deep inguinal lymph node was diagnosed by the pathologist as an internal iliac lymph node metastasis. Six were pathologically proven to be metastasis, five were true positives and 1 was a false negative. The false-negative case was found to have a left obturator lymph node metastasis.

The average contrast agent doses of the bipedal and cervical injections in MR lymphography were 20.75 ml and 13.25 ml, respectively. The homogeneity of variance test F-value and significance (Sig.) value was 0.0 and 1.0, respectively. The average contrast agent doses of the bipedal and cervical injections were not significantly different according to gender. A *t*-test for Equality of Means of the bipedal and cervical images is shown as a t-value (4.361 and 4.361), degrees of freedom (6.000 and 5.981), Sig. (2-tailed) (0.005), Mean Difference (7.500), Std. Error Difference (1.720) and 95%. Confidence intervals of the Difference Lower (3.291 and 3.288) and Upper (11.709 and 11.712) are presented.

The average clearest image time (minutes) of the bipedal and cervical acquisitions in MR lymphography was 29.5 and 9.0, respectively. The homogeneity of variance test F-value and Sig. value was 13.500 and 0.01, respectively. The average clearest image time of the bipedal and cervical acquisitions was significantly different according to gender. A *t*-test for Equality of Means of the bipedal and cervical data is shown: t-value (16.292 and 16.292), degrees of freedom (6.000 and 3.696), Sig.(2-tailed)(0.000), Mean Difference (20.500), Standard Error Difference (1.256) and 95% Confidence interval of the Difference Lower (17.421 and 16.890) and Upper (23.579 and 24.110) is presented.

In our study, the accuracy of interstitial MR lymphography for detecting lymph node metastases was 92.9%. The sensitivity and specificity were 80% (4/5) and 95.7% (22/23), respectively.

**Figure 1:**
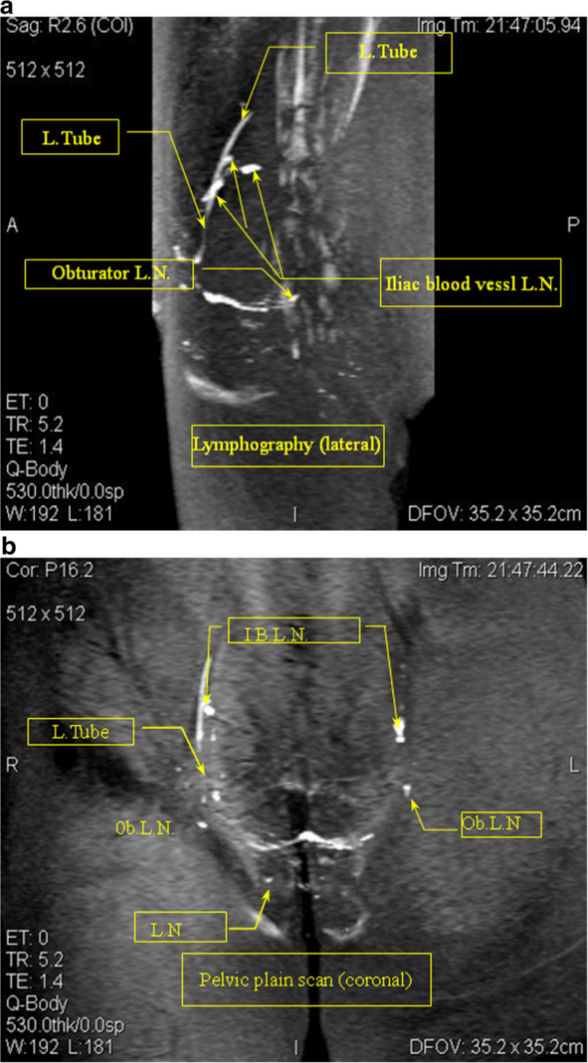
**1–1: MR lymphography (sagittal view) Case 1: A 54-year-old cervical cancer patient.** The MRI scan of the pelvic lymphatic system before lymphography showed no obvious lymph node metastases. The lymphography lateral map shows an irregular shape of the obturator lymph nodes (Ob. L.N.), and the iliac blood vessel lymph nodes (I.B. L.N.) show filling defects. The lymphatic shape was not continuous. MR lymphography in this patient was acquired using a slow subcutaneous injection of 15 ml Gadodiamide and 4 ml 2% lidocaine in the labia majora pudendi followed by a 30 second massage of the injection site. MRI shows the lymph nodes (L.N.) and lymphatic vessels (L.Tube). **1**–**2**: Lymphography (coronal view) Case 1. The contrast with lymphatic (coronal view) irregular shape of the bilateral iliac blood lymph nodes (I.B.L.N.) and the right obturator lymph nodes (Ob.L.N.). A filling defect and the absence of the left inguinal lymph nodes is shown.

**Figure 2:**
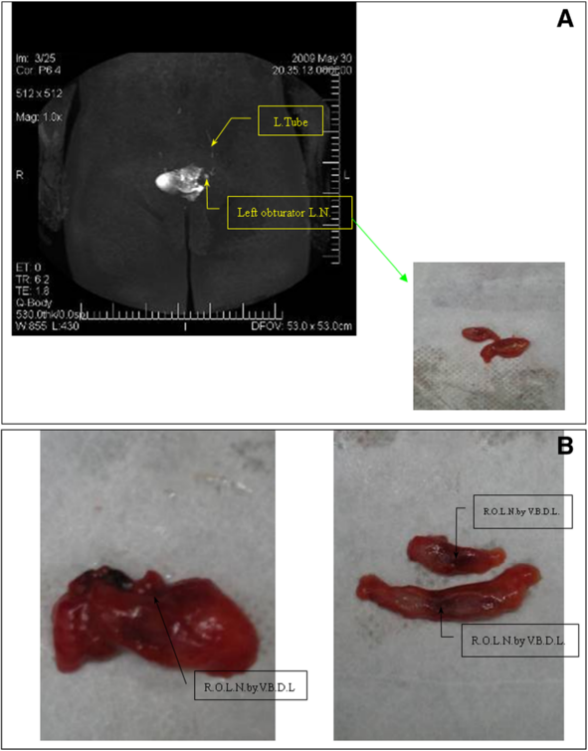
**Pelvic plain scan (axial view) Case 2: MR lymphography of a 47-year-old patient with cervical cancer.** Lymphography (coronal view) Case 2: Coronal MR scans in patients with left obturator lymph nodes are clearly visible, the left side of the developing lyphatic clear portion of the right lymph node imaging. Continuous shaped lymphatic vessels and no abnormal lymph nodes were observed. Surgical specimen of the sentinel lymph node (SLN) (right obturator lymph nodes) (guided by visual blue dye lymphography) Left: SLN (right obturator lymph node (R.O.L.N.) specimens of pathological anatomy (guided by visual blue dye lymphography (V.B.D.L.)). Right: SLN (right obturator lymph nodes) depending on the pathological anatomy of post-mortem specimens can be seen in the lymph nodes guided by visual blue dye lymphography.

**Figure 3:**
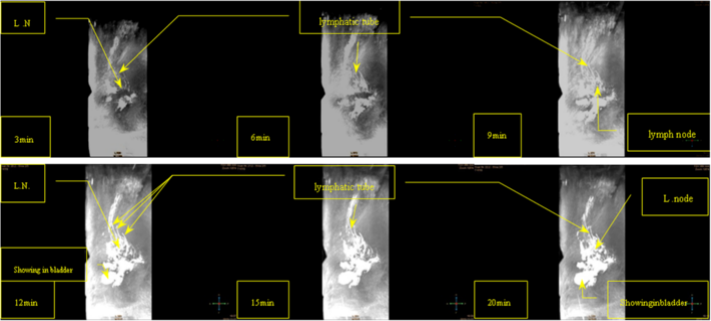
**Lymphography (lateral position) Case3:A 42-year-old cervical cancer patient received a slow injection of 20 ml Gadodiamide and 6 ml 2% lidocaine over three minutes.** The injection was followed by a 20-minute massage within the MRI system. MR lymphography displays the lymph nodes (L.N.) and lymphatic vessels (L.T.) at various time points. After 12 minutes, the contrast agents appeared mainly in the bladder. There are unmarked elliptically shaped lymph nodes of the iliac blood vessel. The lymphography lateral map was clear. There are numerous unmarked areas with similar shapes to the linear lymph vessels.The lymphatic shape was continuous.

**Figure 4:**
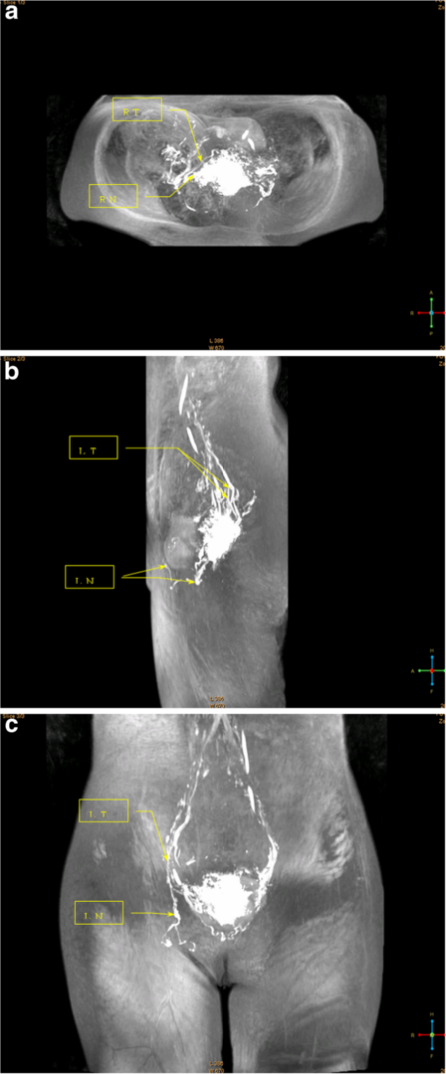
**4–1: Pelvic plain scan (axial view) Case 4: A 38-year-old patient with cervical cancer (Ib1) was injected with Gadodiamide into the cervical tissue.** MR lymphography shows the lymph nodes and lymphatic vessels clearly. MR lymphography in the axial view shows the lymph nodes and lymphatic vessels. The arrow marks the right cervical lymph nodes (R.N.), developing clear, with no abnormal increases and no filling defects. The other arrow displays the continuity of the right small lymphatic channels (R.T.). **4–2**: Lymphography (lateral position). MR lymphography in the coronal view shows the lymph nodes and lymphatic vessels. Two arrows mark the left lymph nodes (L.N.), developing clear, with no abnormal increase and no filling defects. The other arrow displays the continuity of the left small lymphatic channels (L.T.). **4–3**: Pelvis plain scan (coronal view). MR lymphography clearly shows the lymph nodes and lymphatic vessels. The arrow marks the left lymph nodes (L.N.), clearly developed with no abnormal increase and no filling defect. The other arrow displays the continuity of the left small lymphatic channels (L.T.).

All patients tolerated the examination well and no complications or allergic reactions were observed. In the four patients who had bipedal MR lymphography five minutes after the injection of Gadodiamide, the lymphatics and lymph nodes were clearly visible. The image that best displayed the lymph nodes was acquired after 30 minutes. When scanning was extended beyond 45 minutes, image quality decreased and the MR contrast agent was mostly visible in the bladder. In two patients with interstitial MR lymphography, the best image quality in MR lymphography was observed after 17 minutes post-injection (see Case 1, Figure 1). The image quality decreased in these cases after 30 minutes when most of the contrast agent was found in the bladder (see Case 3, Figure 2). In 22 patients with injection of the contrast agent into the cervical tissue, the best image quality occurred nine minutes post-injection (see Case 2 and Case 4, Figures 3 and 4). Cervix lymphography was able to display most cervical lymph node metastases. Interstitial MR lymphography is shown for SLN where Gadodiamide was injected into the cervical tissue in 22 cases (Table 1).

**Table 1:** Results of interstitial MR lymphography, visual blue dye lymphography and pathological anatomy in 22 cases

**case**	**Preoperative MRI**			**Intraoperative blue dye lymphography**		**Pathological anatomy**		
	**position**	**amount**	**nature**	**position**	**amount**	**position**	**amount**	**nature**
1	B.Ob. LN	2	-	B.Ob. LN	2	B.Ob. LN	3	-
2	B.Ob. LN	2	-	B.Ob. LN	2	B.Ob. LN	2	-
3	L.I.I.B.LN	3	-	L.I.I.B.LN	2	L.I.I.B.LN	3	-
4	B.Ob. LN	2	+	B.Ob. LN	1	B.Ob. LN	2	+
5	B.E.I.B.LN.	2	-	B.E.I.B.LN.	3	B.E.I.B.LN.	4	-
6	B.E.I.B.LN.	3	-	B.E.I.B.LN.	2	B.E.I.B.LN.	3	-
7	R.I.I.B.LN	1	+	R.I.I.B.LN	1	R.I.I.B.LN	1	+
8	B.Ob. LN	5	-	B.Ob. LN	2	B.Ob. LN	4	-
9	B.Ob. LN	2	-	B.Ob. LN	2	B.Ob. LN	2	-
10	R.Ob. LN	1	-	R.Ob. LN	1	R.Ob. LN	1	-
11	L.Ob. LN	2	-	L.Ob. LN	1	L.Ob. LN	3	-
12	L.D.I.LN.	2	-	L.I.I.B.LN	1	L.I.I.B.LN	3	-
13	L.Ob. LN	1	-	L.Ob. LN	1	L.Ob. LN	1	+
14	B.Ob. LN	3	-	B.Ob. LN	1	B.Ob. LN	4	-
15	C.LN.	4	-	C.LN.	2	C.LN.	4	-
16	L.Ob. LN	3	-	L.Ob. LN	1	L.Ob. LN	3	-
17	R.Ob. LN	3	+	R.Ob. LN	3	R.Ob. LN	4	+
18	L.Ob. LN	3	+	L.Ob. LN	3	L.Ob. LN	3	+
19	L.Ob. LN	3	+	L.Ob. LN	1	L.Ob. LN	3	+
20	B.E.I.B.LN.	2	-	B.E.I.B.LN.	3	B.E.I.B.LN.	4	-
21	B.Ob. LN	3	-	B.Ob. LN	2	B.Ob. LN	5	-
22	B.E.I.B.LN.	4	-	B.E.I.B.LN.	3	B.E.I.B.LN.	4	-
Total		56			40		66	

(−) Negative case was diagnosed as lymph node metastasis. (+)Positive case was diagnosed as lymph node metastasis.

(L.) Left, (R.)Right, (B.) Bilateral, (Ob. LN.) obturator lymph nodes, (E.I.B.LN.) external iliac blood vessel lymph nodes, (I.I.B.LN.) internal iliac blood vessel lymph nodes, cervical lymph nodes (C.LN.), deep inguinal lymph node (D.I.LN.).

In 22 cases, SLN were observed in operations guided by blue dye lymphography where visual blue dye was injected into the cervical tissue. Tracer of blue dye travels along the same lymphatic drainage pathway as cervical cancer cells to reach the first station lymph nodes. In surgery, SLN can be identified according to the stained lymph nodes, and can help to guide the biopsy. The blue dye tracer method helped to identify 40 SLN in 22 patients. In our study, intraoperative blue dye lymphography showed an SLN average of 1.8 per patient (40/22) (see Table 1).

Preoperative MRI and intraoperative blue dye lymphography were compared: MRI lymphography showed an accuracy rate of 85% for the detection of SLN (56/66). Tracer of blue dye lymphography showed an accuracy rate of 61% for the detection of SLN (40/66). A significance of p < 0.01 was calculated using a *χ*^2^-test.

Preoperative MRI and pathological anatomy were also compared: depending on the pathological anatomy of the specimens, 66 positive SLN could be found (guided by preoperative MRI and visual blue dye lymphography), on average, 3.0 lymph nodes per patient (66/22) were observed.

## Discussion

Interstitial MR lymphography in cervical cancer allows for the detection of lymph node metastases non-invasively. Interstitial MR lymphography is gradually attracting more attention since it helps to overcome the deficiencies of standard MR imaging techniques in making qualitative diagnoses of lymph node status. MR lymphography is currently the only way to observe lymph nodes and to understand their function and is of great value in diagnosing the involvement of retroperitoneal and pelvic lymph nodes.

Lymphatic metastasis is the primary metastasis occurring in cervical cancer, which not only determines the prognosis but also the choice of treatment and surgical methods. At present, the various methods for detecting lymph node metastases, such as CT, MR and PET-CT, all have low sensitivity and specificity. Recently, lymphography has become the primary focus in detecting lymph node metastases. Direct lymphography involves injecting contrast agents (such as iodized oil) subcutaneously, which requires observation of lymphatic metastases under X-ray. This technique is time-consuming and potentially dangerous, with incidents of pulmonary embolisms reported. Furthermore, the spatial resolution of X-ray lymphography is poor, limiting its use in clinical practice. Interstitial MR lymphography with subcutaneous injection of contrast agents (strictly controlled in the skin) allows scanning of the pelvic lymph system. This method is a good way to visualize the pelvic lymph nodes, and has been confirmed by recent research studies [4,5].

A prospective consecutive study for SLN identification with preoperative SPECT/CT and planar images (technetium-99 m colloid albumin injection around the tumor) and posterior intraoperative detection with both blue dye and a handheld or laparoscopic gamma probe was reported by Díaz-Feijoo et al. A total of 35 SLN were detected with planar images and 40 SLN were identified and well-located by SPECT/CT lymphoscintigraphy (median 2.0 nodes per patient). In 5/22 patients (22.7%) SPECT/CT procedure improved the number of localized SLN. Intraoperatively, 57 SLNs were identified, with a median of 3 SLNs per patient by gamma probe (a total of 53 hot nodes) and a median of 2 nodes per patient after blue dye injection (a total of 42 blue nodes). Microscopic nodal metastases (eight nodes, corresponding to four patients) were confirmed in 18 [6]. Likewise, in a study by Lécuru F et al., intraoperative radioisotope-blue dye mapping detected at least one SLN in 136 patients (97.8%; 95% CI, 93.8% to 99.6%), 23 of whom had true-positive results and two who had false-negative results, yielding 92.0% sensitivity (23 of 25; 95% CI, 74.0% to 99.0%) and 98.2% NPV (111 of 113; 95% CI, 74.0% to 99.0%) for node metastasis detection. No false-negative results were observed in the 104 patients (76.5%) in whom SLN were identified bilaterally [7]. In our study, the accuracy of interstitial MR lymphography for detecting lymph node metastases was 92.9%. The sensitivity and specificity were 80% (4/5) and 95.7% (22/23), respectively. The PPD was 100%, and NPD was 95.7%. The results before surgery non-invasively were superior in detecting lymph node metastases compared to the literature.

Therefore, interstitial MR lymphography in detecting lymph node metastases is of significance for clinical decision making in patients with cervical cancer. MR lymphography should be routinely performed prior to surgery since it can often identify new findings that are usually missed by clinical examinations, thus making diagnosis and staging more accurate.

## Conclusion

Interstitial MR lymphography enables the visualization of the extent and shape of pelvic lymphatic vessels and lymph node metastases in patients with cervical cancer. The evaluation of lymph node metastases is important for the treatment and prognosis of cervical cancer.

## Competing interests

The authors declare that they have no competing interests.

## Authors’ contributions

YH: corresponding author, conceived and designed the project. She performed specific operations, analyzed data, and provided materials and wrote the final paper. She is responsible for this project. LX: participated in the recent part of the collection of data. JF: participated in the recent part of the collection of data. YH: provided supervision for the project. ZZ: contributed to MR imaging. HY: contributed to MR imaging. CZ: participated in the most recent data collection. BZ: contributed to MR imaging. All authors read and approved the final manuscript.

## Financial support

This study was supported by Health Bureau of Nanjing(No.ZKX08021).

## Pre-publication history

The pre-publication history for this paper can be accessed here:

http://www.biomedcentral.com/1471-2407/12/360/prepub

## References

[B1] MacArthur GJ Wright M Beer H Paranjothy S Impact of media reporting of cervical cancer in a UK celebrity on a population-based cervical screening programme J Med Screen 2011 18 4 204 209 Epub 2011 Dec 7. PubMed PMID:22156146 10.1258/jms.2011.011092 22156146

[B2] Zhao FH Tiggelaar SM Hu SY Xu LN Hong Y Niyazi M Gao XH Ju LR Zhang LQ Feng XX Duan XZ Song XL Wang J Yang Y Li CQ Liu JH Liu JH Lu YB Li L Zhou Q Liu JF Zhao N Schmidt JE Qiao YL A multi-center survey of age of sexual debut and sexual behavior in Chinese women: Suggestions for optimal age of human papillomavirus vaccination in China Cancer Epidemiol 2012 36 4 384 390 Epub 2012 Feb 27. PubMed PMID: 22377277 10.1016/j.canep.2012.01.009 22377277 PMC5523958

[B3] Bézu C Coutant C Ballester M Feron J-G Rouzier R Uzan S Daraï E Ultrastaging of lymph node in uterine cancers J Exp Clin Cancer Res 2010 29 5 10.1186/1756-9966-29-5 20092644 PMC2828991

[B4] Lohrmann C Felmerer G Foeldi E Bartholomä JP Langer M MR lymphangiography for the assessment of the lymphatic system in patients undergoing microsurgical reconstructions of lymphatic vessels Microvasc Res 2008 76 1 42 45 Epub 2008 Mar 20. PubMed PMID: 18456290 18456290 10.1016/j.mvr.2008.03.003

[B5] Vilarino-Varela MJ Taylor A Rockall AG Reznek RH Powell ME A verification study of proposed pelvic lymph node localisation guidelines using nanoparticle-enhanced magnetic resonance imaging Radiother Oncol 2008 89 2 192 196 Epub 2008 Sep 2. PubMed PMID: 18771811 18771811 10.1016/j.radonc.2008.07.023

[B6] Díaz-Feijoo B Pérez-Benavente MA Cabrera-Diaz S Gil-Moreno A Roca I Franco-Camps S Fernández MS García-Jiménez A Xercavins J Martínez-Palones JM Change in clinical management of sentinel lymph node location in early stage cervical cancer: the role of SPECT/CT Gynecol Oncol 2011 120 3 7 Epub 2011 Jan 6. PubMed PMID: 21215440 10.1016/j.ygyno.2010.12.336 21215440

[B7] Lécuru F Mathevet P Querleu D Leblanc E Morice P Daraï E Marret H Magaud L Gillaizeau F Chatellier G Dargent D Bilateral negative sentinel nodes accurately predict absence of lymph node metastasis in early cervical cancer: results of the SENTICOL study J Clin Oncol 2011 29 13 1686 1691 Epub 2011 Mar 28. PubMed PMID 21444878 10.1200/JCO.2010.32.0432

